# Reconstruction of Relative Phase of Self-Transmitting Devices by Using Multiprobe Solutions and Non-Convex Optimization

**DOI:** 10.3390/s21072459

**Published:** 2021-04-02

**Authors:** Rubén Tena Sánchez, Fernando Rodríguez Varela, Lars J. Foged, Manuel Sierra Castañer

**Affiliations:** 1 Information Processing and Telecommunications Centre, ETSI Telecomunicación, Universidad Politécnica de Madrid, 28040 Madrid, Spain; rubents93@gr.ssr.upm.es (R.T.S.); f.rodriguezv@upm.es (F.R.V.); 2 Microwave Vision Group, Pomezia, 00071 Rome, Italy; lars.foged@mvg-world.com

**Keywords:** antenna measurements, phase reconstruction, post-processing, multiprobe, reference-less

## Abstract

Phase reconstruction is in general a non-trivial problem when it comes to devices where the reference is not accessible. A non-convex iterative optimization algorithm is proposed in this paper in order to reconstruct the phase in reference-less spherical multiprobe measurement systems based on a rotating arch of probes. The algorithm is based on the reconstruction of the phases of self-transmitting devices in multiprobe systems by taking advantage of the on-axis top probe of the arch. One of the limitations of the top probe solution is that when rotating the measurement system arch, the relative phase between probes is lost. This paper proposes a solution to this problem by developing an optimization iterative algorithm that uses partial knowledge of relative phase between probes. The iterative algorithm is based on linear combinations of signals when the relative phase is known. Phase substitution and modal filtering are implemented in order to avoid local minima and make the algorithm converge. Several noise-free examples are presented and the results of the iterative algorithm analyzed. The number of linear combinations used is far below the square of the degrees of freedom of the non-linear problem, which is compensated by a proper initial guess. With respect to noisy measurements, the top probe method will introduce uncertainties for different azimuth and elevation positions of the arch. This is modelled by considering the real noise model of a low-cost receiver and the results demonstrate the good accuracy of the method. Numerical results on antenna measurements are also presented. Due to the numerical complexity of the algorithm, it is limited to electrically small- or medium-size problems.

## 1. Introduction

Near-field (NF) characterization of the radiation of devices requires, in general, to acquire both the amplitude and phase information of the radiated fields [1]. The transformation from the spatial domain of the field distribution to the spectral domain of propagating waves requires the measurement of the amplitude and phase information. In order to do that, usually a reference channel is used, so that the signal that feeds the device under test (DUT) is used as a reference to retrieve the amplitude and relative phase between measurement points.

The challenges derived from the development of technologies like 5G [2], integrated devices from internet of things (IoT) [3], or the characterization of electromagnetic compatibility (EMC) [4], are examples of applications in which the measurement of the radiation cannot be done in a conventional manner. In 5G, the complexity arises from the necessity of integration derived from the development of multiple-input multiple-output (MIMO) or smart antennas along with others. In [5], a description of the challenges for on-chip antennas (OCA) is analyzed. This overview highlights the difficulties found when measuring devices where there is not a radio frequency (RF) connector due to physical limitations or because it would be cost-ineffective. Another example can be seen in the development of wireless communications. The improvement of the technology and the necessity of massive amounts of data transmission is leading to massive wireless sensor networks of which characterization is not trivial [6,7]. To guarantee reliability of the network, it is important to ensure the radiation performance of the single elements. The characterization of the radiation of these devices is not straightforward, since the energy source is inside the device and the reference channel is not accessible. In this paper, we refer to these devices as self-transmitting devices.

There are useful solutions in the literature that represent an alternative to classical methods that are based on amplitude and phase measurements. Most of these techniques are based on amplitude-only methods to retrieve the relative phase between measurement points. Examples of this can be seen in iterative methods between two surfaces [8,9,10], holographic techniques [11,12,13], or interferometry [14]. The downside of these techniques is that either they are time or cost-inefficient. Besides that, in general, the phase retrieval methods lead to a non-linear, non-convex optimization problem [15]. It is well known that the inherent serious problem of these optimization methods is the high occurrence of local minima. A good practice is to find a proper initial guess, as was demonstrated in [16]. Nevertheless, sometimes it is not easy to find a proper initial guess that compensates the lack of phase information, and the techniques will generally not find a unique solution. 

There are some phase retrieval solutions in the literature that exploit the correlation between measurement probes over the same surface, like the approaches followed in [16,17]. In these works, an interferometric solution was proposed by combining the signal received by two identical probes that move on a single surface. The only complexity of the solution is the combiner network. However, there are some drawbacks inherent to the way the phase is retrieved, like propagation errors or the necessity of changing the separation between probes for different frequencies to make it time-efficient and still satisfy Nyquist criteria. Moreover, in the interferometric solution, the measurement surface is divided in subsets, so that phase unknowns are introduced. The way this problem was addressed was by using a non-redundant representation of the field.

In [18], random linear combinations between signals on the same measurement surface were analyzed. It was found that using partial knowledge of the phase differences between measurement points could provide enough information but only if a sufficient number of linear combinations between signals is computed. A similar approach was followed in [19], but in this case by sampling the surface with special probes. It was demonstrated that the amount of useful information that can be retrieved from special probes measuring on the same surface can be more than measuring on different surfaces. The numerical simulations showed that the reconstruction is sensitive to noise and propagation errors, since large errors were observed for some positions where the phase error expected for consecutive points should be small according to the noise-free scenario. Therefore, the results show that the lack of phase information can be compensated if sufficient information of the partial correlation of signals over the same measurement surface is known. 

In this paper, a novel approach based on taking advantage of the intrinsic characteristics of multiprobe systems based on rotating arch [20] is presented. Spherical NF multiprobe measurement systems are solutions that provide low acquisition times and are appropriate to reduce the measurement time for characterizing self-transmitting devices. The solution proposed is based on using the top probe of spherical multiprobe systems as a reference channel and deals with the retrieval of phase unknowns that are introduced in scenarios where the multiprobe arch rotates, and consequently the reference antenna is not always on-axis.

**Figure 1:**
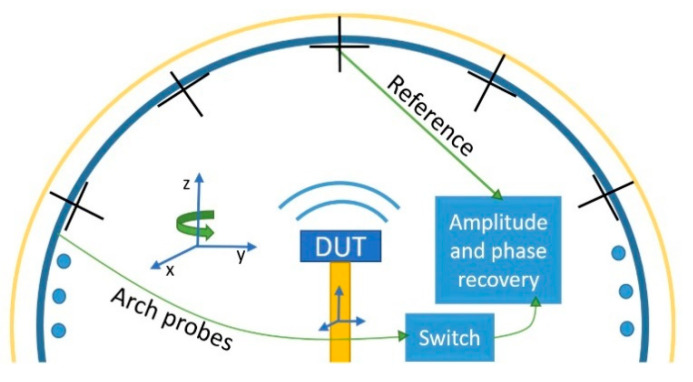
Multiprobe set-up for phase retrieval.

This situation where a self-transmitting device is being measured using as reference the top probe of the measurement arch, was already considered in [21]. In this paper, the top probe was used as a reference signal to retrieve the relative phase between measurement points, see Figure 1. However, this technique was only useful for scenarios where the multiprobe arch is static, being able to use always the same fixed reference antenna.

The implementation of the solution based on the fixed on-axis probe provides a good reference channel, but this reference is lost if the top probe is rotated. In this paper, a solution to this problem is proposed. Generally, a multiprobe measurement system is based on rotating arches for spherical NF measurements, as depicted in Figure 1. In the figure, the multiprobe array consists of a group of evenly spaced dual polarized elements (depicted as crosses) along a circumference that composes a 2D cut of the measurement surface. The whole measurement surface is sampled by rotating the DUT in azimuth, and the arch in elevation.

The generic case of a multiprobe measurement system is analyzed in this paper, where the multiprobe arch rotates in elevation, in order to increase the number of samples. For this situation, a reconstruction of the virtual reference antenna (top probe) is needed, since it is not static anymore. If these scenarios are considered, phase unknowns are introduced in the system, since for every arch rotation of the array, there is a change in the reference channel. Thus, it is necessary to develop a procedure for retrieving these phase unknowns.

In Section 2, the phase reconstruction algorithm is presented from the mathematical point of view. Then, Section 3 shows some noise-free simulations for different sizes of antennas and schemes with rotation of the multiprobe arch. In Section 4, a real receiver noise model is included in the simulations, and the reconstruction results are analyzed in the NF and far-field (FF). Some measurement results from a spherical multiprobe system will be shown in Section 5. Finally, in Section 6, the major conclusions of the proposed method are presented.

The main contribution of this paper is the reconstruction of the radiated phase of devices where the RF reference is not accessible. This is done by virtue of an iterative algorithm to recover the phase coherence between measurement points when the arch of multiprobe systems is rotated in elevation. The algorithm exploits the correlated information that can be obtained by combining multiprobe measurement systems and post-processing techniques. The solution herein proposed integrates multiprobe measurement systems with the degree of freedom of elevation rotations, and low-cost receivers. Thus, it provides a cost-effective and fast solution applicable to typical 5G, IoT, or EMC devices. 

## 2. Phase Reconstruction Algorithm

This section explains the algorithm to reconstruct the phase when the arch rotates and the reference antenna is not fixed. First, the Spherical Wave Expansion (SWE), the field basis representation used to solve the phase retrieval algorithm is presented. Because the phase reconstruction is a nonlinear algorithm highly dependent on the choice of the initial point, a procedure for the computation of such initial guess will be shown in a following section. Finally, an iterative optimization algorithm for the phase reconstruction will be introduced. In this case, it will be seen that the strategy to get the phase unknowns is based on the division of the measured data into different subsets. The first subset, corresponding to the reference probe always on-axis, will constitute the initial guess of the algorithm. 

### 2.1. The Spherical Wave Expansion

Let us consider a spherical coordinate system with the DUT located on the origin. The SWE allows to expand the field radiated by the DUT in a set of orthonormal basis functions weighted by coefficients:

(1) $$\;\;\;\;\;\;\;\;\;\;\;\;\;\;\;\;\;\;\;\;\;\;\;\;\;\;\;\;\;\vec{E}\left(r,\theta ,\varphi \right)=\overset{2}{\underset{s=1}{\sum }}\overset{N}{\underset{n=1}{\sum }}\overset{n}{\underset{m=-n}{\sum }}Q_{smn}{\vec{F}}_{smn}^{\left(3\right)}\left(r,\theta ,\varphi \right)\;\;$$

(r,θ,φ) being spherical coordinates, $Q_{smn}$ the Spherical Wave Coefficients (SWC), and ${\vec{F}}_{smn}^{\left(3\right)}$ the spherical wave basis functions. N is the truncation number of the expansion which can be estimated by the following rule of thumb:

(2) $$\;N=\lceil kr_{0}\rceil +10$$

where $r_{0}$ is the radius of the minimum sphere and the brackets indicate the largest integer smaller than or equal to the number inside them.

The truncation number sets a limit on the minimum number of samples required for an accurate NF to FF transformation. This number of samples is given as a function of the angular increments $\Delta \theta =\Delta \varphi =\frac{\pi }{N}$. This is a traditional criterion employed for conventional complex measurements and it will be used here.

Equation (1) admits a matrix-based representation when the SWE is evaluated on discrete points, i.e., the measurement samples of near-field range:

(3) w=Cq

where w and q are vectors containing the measured samples and SWC, respectively, and C a coupling matrix which performs the necessary computations. Because w is known from the measurements, q can be computed by inverting (2), which can be done calculating the pseudoinverse of C. However, only the amplitude information from w is available so an alternative approach must be followed: a phase retrieval algorithm based on an iterative optimization.

### 2.2. Initial Guess Computation 

**Figure 2:**
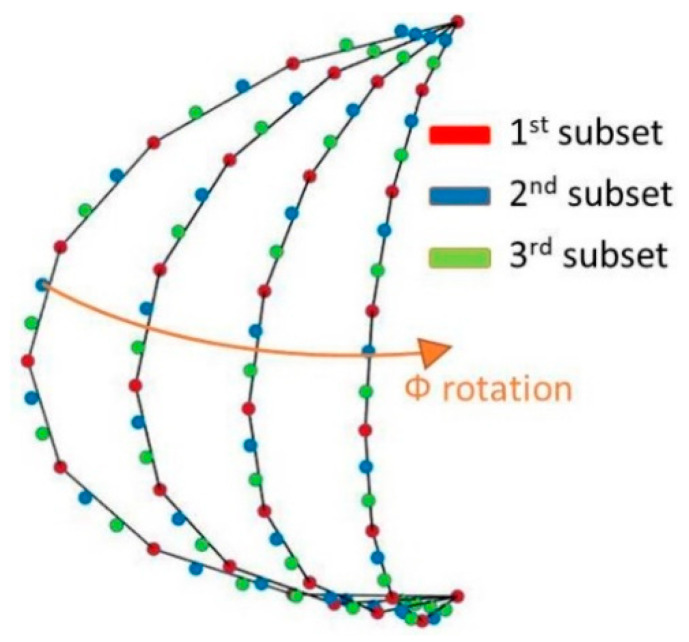
Sampling scheme and division in subsets, N = 3.

When the multiprobe arch rotates, the measured electromagnetic field can be divided into N different subsets, depending on the position of the measurement arch. A simplified description of the situation can be seen in Figure 2, where for the sake of simplicity, only three subsets are considered. According to [21], it is possible to retrieve the relative phase between points that belong to the subset where the top probe is in the center of the arch, first subset from now on. The method employed to retrieve the relative phase between points is based on Ludwig’s third definition [22] of polarization for the top probe. In that sense, the polarization properties of the electromagnetic field are followed for different azimuth positions. The polarization properties of the electromagnetic field for the top probe can be used in order to determine the phase unknowns of the first subset where the on-axis probe is not displaced.

If the previous post-processing technique is applied, it can be assumed that ideally, the first subset is properly reconstructed in amplitude and phase. For the other N − 1 subsets, only for each azimuth cut, the amplitude and the relative phase between probes are known, because the probe that was formerly on the top axis can still be used as a common reference between all samples of the given cut. Thus, for each subset, there are M phase unknown terms corresponding to the relative phases between the different azimuth positions. These remaining phase unknowns are described in Equation (4), where i = 1, 2, …, M, with M representing the number of azimuth cuts. Thus, for every subset different from the first one, the number of phase unknowns will be M. The total number of phase unknowns will be equal to M times (N−1).

(4) $$\tilde{E}{\left(S_{j}\right)}_{i}=E{\left(S_{j}\right)}_{i}e^{\varphi ij},i=1,\ldots ,M\;\mathrm{and}\;j=2,\ldots ,\;N$$

The information of the first subset can be used to compute an initial set of spherical coefficients Qinit. Depending on the sampling requirements of the DUT, this computation will have errors due to inaccuracy in the coefficient calculation because of the down-sampling of the input field in the elevation dimension. The criticality of this initial guess is clear since it will determine a good convergence of the algorithm. Nevertheless, as it was stated in the introduction, it is not critical for characterizing the radiation of electrically small devices.

### 2.3. Optimization and Linear Combinations 

It is necessary to develop an algorithm to include all the a priori information of the radiation of the device into the problem, so that the convergence is guaranteed. Since the complex measurements are only known for the first subset, it is necessary to work only with the magnitude of the fields and solve the inverse problem described in Equation (5), which corresponds to a common phase retrieval problem [23].

(5) |Ax|=|b|

where |b| and x are vectors containing the amplitude information of the measured samples and the DUT spherical wave coefficients, respectively. The coupling matrix containing the necessary terms to model the interaction between the coefficients and the near-field samples is represented by A. 

It is well known that the phase shift between two complex signals can be determined by a set of four squared magnitude measurements [16], which includes the in-phase and in-quadrature combination of the signals under interest. Thereby, this mathematical relation between the measured signals can be implemented for the complex values of each azimuth cut from subset 2 to N. The linear combination of the signals where the relative phase is measured can be computed and added to the optimization algorithm. The reason for choosing this approach is that the linear combination of the measured signals can be easily modeled with the introduced matrix notation as demonstrated in [18]. This constitutes an efficient way of incorporating the partial knowledge we have about the phase of the measured field into our phase retrieval problem. 

**Figure 3:**
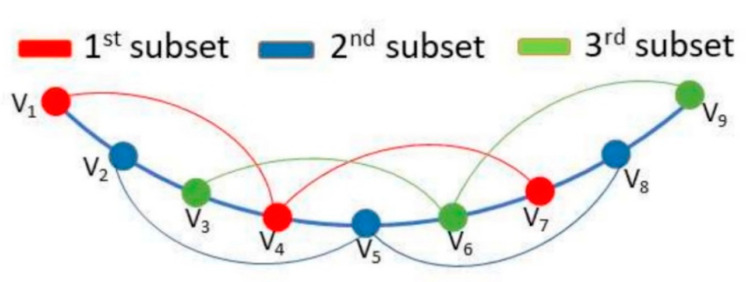
Signals combination for oversampling equal to 3, one azimuth cut.

This linear combination will be done for both E_θ_ and E_φ_ components, in order to extract also the relative phase between the measured points for the two orthogonal polarizations. Then, for each subset and azimuth cut, a total number of N_θ_ − 2 points are combined. Since the in-phase and in-quadrature combinations are needed, a total number of 2(N_φ_(N_θ_ − 2)) rows are included into the A matrix for each subset. The number of rows added due to the combination between the phase difference between E_θ_ and E_φ_ is 2N_φ_N_θ_. In Figure 3, a representation of the voltage combination performed for three different angular positions of the multiprobe arch can be seen. These voltages represent the combination of E_θ_ or E_φ_ components of the field. For instance, assuming that we want to incorporate the relative phase information between E_θi_ and E_θk_ belonging to the same subset, Equation (6) shows the combinations that will be used to include the information in the optimization algorithm.

(6) $$\left(\begin{array}{c} \begin{array}{c} b1\\ b2 \end{array}\\ \begin{array}{c} b3\\ b4 \end{array} \end{array}\right)=\left(\begin{array}{c} \begin{array}{c} E\theta i\\ E\varphi k \end{array}\\ \begin{array}{c} E\theta i+E\varphi j\\ E\theta i+jE\varphi k \end{array} \end{array}\right)=\left(\begin{array}{c} \begin{array}{c} 1\;\;\;\;\;\;\;0\\ 0\;\;\;\;\;\;\;\;1 \end{array}\\ \begin{array}{c} 1\;\;\;\;\;\;\;1\\ 1\;\;\;\;\;\;\;j \end{array} \end{array}\right)\left(\begin{array}{c} E\theta k\\ E\varphi k \end{array}\right)$$

To do that in a systematic way, the measurements and the coupling matrix can be multiplied by another matrix P as shown in Equation (7), so that the linear combination between rows is performed. In the matrix, I refers to the identity matrix that accounts for the measurements themselves, whereas $LC_{i}$ refers to a matrix which performs the linear combinations in a similar way as shown in Equation (6). Thus, the matrix P will consider the in-phase and in-quadrature combination between subsets and different polarizations. By using this approach, the number of degrees of freedom is not squared as it was stated in [18], but the phase variations of subsets in combination with a good initial guess will make the iterative algorithm converge to an accurate solution as will be shown in the numerical results.

(7) $$P=\left[\begin{array}{c} I\\ LC_{1}\\ .\\ .\\ .\\ LC_{N} \end{array}\right]$$

The optimization problem can be finally formulated as described by Equation (8).

(8) $$\underset{x\in C^{m}}{\min}|\;\left(\left|\left|PAx\right|-\left|Pb\right|\right|\right){|}^{2}\;\;$$

It is important to stress that the procedure described herein is different from phaseless approaches, since the phase is retrieved partially thanks to the multiprobe system and top probe solution as a reference channel. This provides a proper initial guess as will be seen in the following sections.

### 2.4. Iterative Optimization 

To make the algorithm converge to a proper solution, it is necessary to apply an iterative process. The basic idea of the iterative process rests on the assumption that the initial guess error will be larger for high order modes of the spherical wave expansion, and therefore the algorithm will tend to fall into local minima solutions with larger errors in this part of the spectrum. 

**Figure 4:**
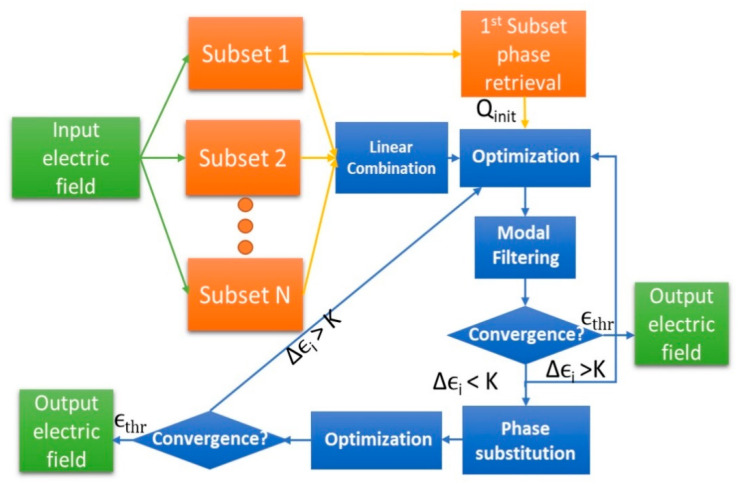
Iterative optimization algorithm.

To solve this problem, an iterative scheme can be implemented as depicted in Figure 4. The error, which is the output of the cost-function, is computed as stated in Equation (9),

(9) $$\epsilon_{i}={\left|\left|{\left|Ax_{i}\right|}^{2}-{\left|b\right|}^{2}\right|\right|}_{2}$$

where ${\left|\right|}_{2}$ denotes the two-norm and $x_{i}$ the retrieved vector of coefficients in the ith iteration. The error threshold, $\varepsilon_{thr}$, defines the stopping condition of the algorithm. The decisions of the iterative algorithm depend on the computation of the incremental error $\Delta \varepsilon_{i}$, which is calculated as described by Equation (10).

(10) $$\Delta \epsilon_{i}=\frac{\epsilon_{i}}{\epsilon_{i-1}}$$

Let us assume without loss of generality that the iterative process is in the *i*th iteration. Depending on the evolution of $\Delta \varepsilon_{i}$, the algorithm can either continue iterating, stop because the threshold is reached, or because there is saturation between iterations (represented by small values of $\Delta \varepsilon_{i}$). If the algorithm continues, it can either apply modal filtering [24] or phase substitution of the known relative phases within the same subsets and azimuth cuts. This will help the algorithm to find a better initial guess for every iteration. 

The modal filtering is implemented considering the empirical condition established by Equation (2). The practical implementation of the algorithm is based on filtering out from a mode index that is smaller than the criteria defined by Equation (2), that is N_filter_ < $N_{SWE}$. It is assumed that the error of these modes is large. Consequently, this regularization helps the algorithm to find solutions where the energy of the modes larger than N_filter_ is reduced. This procedure allows to avoid local minima. Nevertheless, it will limit the accuracy of the solution found, since the energy of some modes that are still relevant to reconstruct the field are set to zero in the iterative process. In that case, saturation is reached and increasing the cut-off frequency (N_filter_) for the next iterations improves the accuracy of the solution found. 

This filtering technique in combination with phase substitution of the known information provides a proper initial guess between iterations that tends monotonically to the solution as described in the following sections.

### 2.5. Comparison with Pure Phaseless Approaches

The introduced method constitutes an intermediate solution between the standard phase retrieval approaches and reference-less measurement techniques. The top probe works as a reference channel, but when it is moved, this reference is lost, so only partial information of the phase is obtained. Therefore, the use of standard phaseless methods is required too. The introduced iterative optimization algorithm works with pure amplitude-only data. It is well known that phaseless techniques are nonlinear and prone to get trapped in local minima. The lack of phase must be compensated with additional information such as multiple surfaces [25], oversampling, or additional hardware [18]. This usually increases the measurement time, which makes phaseless measurements an inconvenient and costly alternative. In this case, the additional information is incorporated into the problem as a partial phase relationship between the measurement samples. The linear combinations and initial guess are the means to introduce this partial phase information. No additional time is required to retrieve this partial phase because only one spherical surface is scanned with the same angular increments of traditional measurements. Because the number of unknown phase relationships is relatively low compared to the available of phase information, the phaseless algorithm shows good performance obtaining low convergence errors. This will be shown in the next section by means of numerical simulations and antenna measurement examples. 

## 3. Noiseless Numerical Simulations

It was introduced above that the computational cost of the solution due to large dimensions of the coupling matrix make it not suitable for electrically large problems. Thus, electrically small and medium problems will be analyzed in this section for the case of noise-free measurements. 

**Figure 5:**
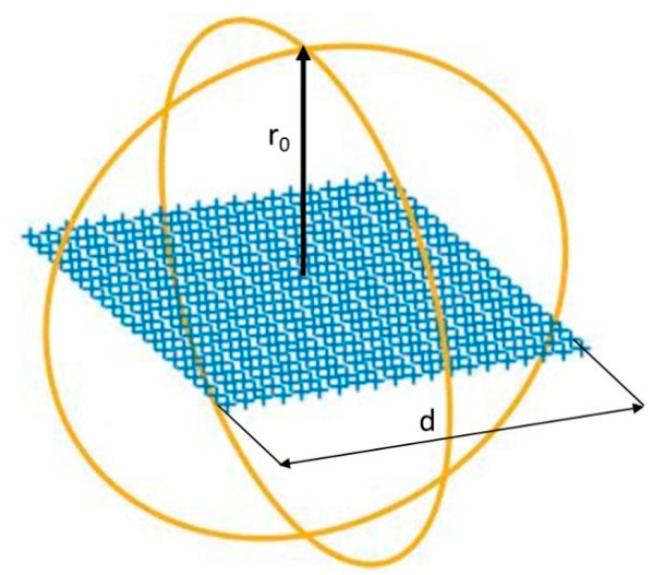
Simulated model of DUT for different electrical size.

It is assumed that the phase retrieval based on Ludwig’s third definition vectors is ideal. Thereby, the phase from the first subset is retrieved without errors. For the sake of simplicity, the same geometry of the DUT will be used to perform the different simulations. A representation of the DUT can be seen in Figure 5. The radiator consists of a set of equally spaced uniformly excited electric Hertzian dipoles. The number of dipoles is kept fixed, and equal to 20. The parameters r_0_ and d are changed for different simulations. In these numerical simulations, full range in azimuth will be used so that φ ∈ [0, 2π] and θ ∈ [0, π]. To simulate a more realistic case, some y-component excitation (below −30dB with respect to the x-component) was introduced by adding y-polarized dipoles in the same position and in phase with the x-polarized dipoles.

**Figure 6:**
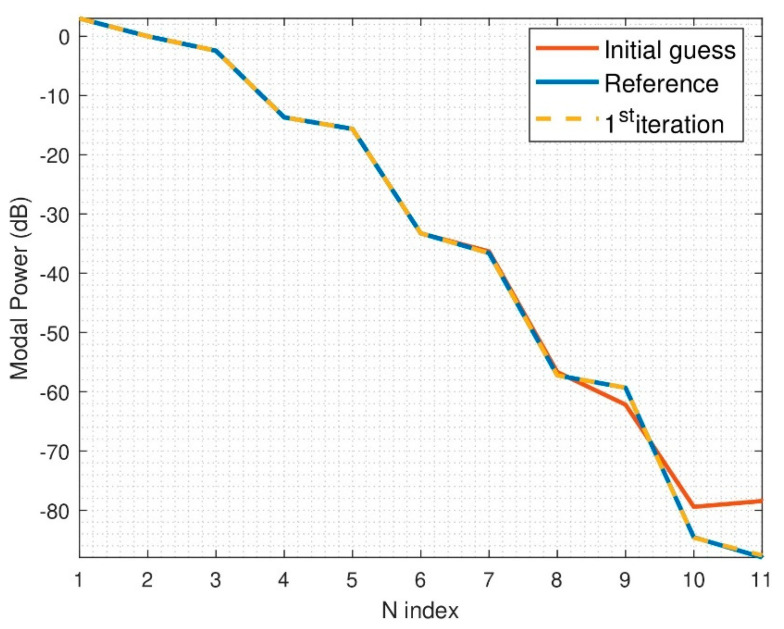
N-power spectrum of reconstructed field, r_0_ = 4λ, d = λ, 2 arch positions.

Let us analyze the performance of the algorithm for an electrically small problem, particularly when d = λ and r_0_ = 4λ. The step needed in θ and φ is 12.4°. For this simulation, it is assumed that the angular step between probes of the multiprobe system is 22.5°, which means that two multiprobe arch positions are necessary since the sampling step used will be 11.25°, giving as a result two subsets. For this particular case, it was decided that each iteration of the algorithm encompassed 600 iterations in the optimizer. The N-power spectrum of the spherical wave coefficients can be seen in Figure 6. In this case, the field variations are smooth, thus, the initial guess is close to the global minimum and only one iteration of the optimization algorithm is necessary to converge (600 iterations). Although not presented here, it was tested that with a random or zero initial guess, the algorithm is not able to find the proper solution, since there is a lack of information of the phase variations between azimuth cuts that the initial guess of the proposed algorithm provides.

**Figure 7:**
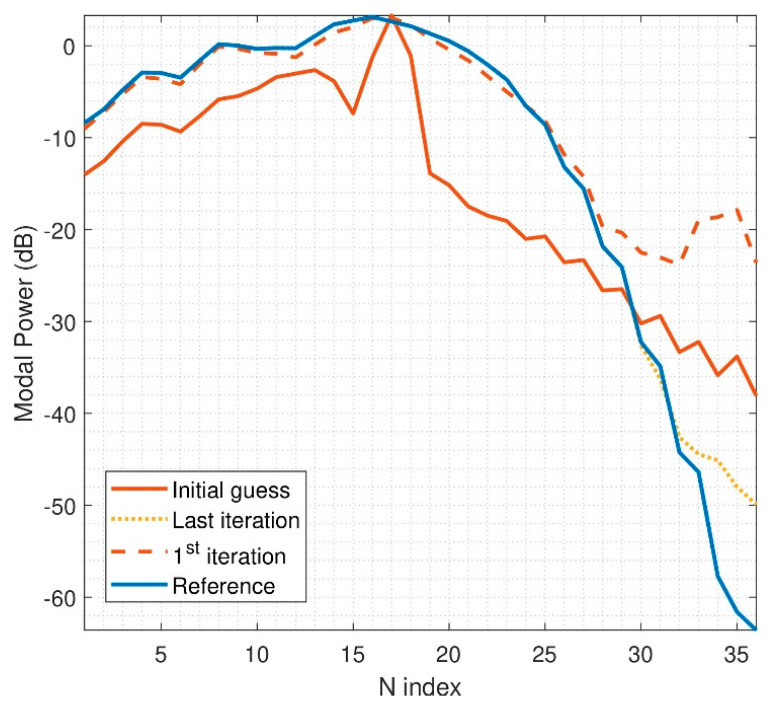
N-power spectrum of reconstructed field, r_0_ = 8λ, d = 6λ, 2 arch rotations.

If an electrically medium problem is analyzed, the radiation of the device will not be that smooth, as in the previous example. In the following case, the size of the DUT will be fixed to d = 6λ and r_0_ = 8λ. The step needed in θ and φ is 6°. The step between measurement probes of the multiprobe system is considered to be 10°. Thus, two multiprobe arch positions are needed. The problem represented here is more challenging in terms of computational resources; actually, the matrix *P* has 20.448 rows. The initial guess of the iterative algorithm as well as the retrieved solution can be seen in Figure 7. It is observed that the initial guess is contaminated by the spectrum aliasing produced by the down-sampling of the initial guess computation. However, the iterative algorithm is able to find an accurate solution. Naturally, the results cannot be as good as the ones obtained for an electrically small radiator, but there is a very good agreement after the optimization algorithm is applied.

**Figure 8:**
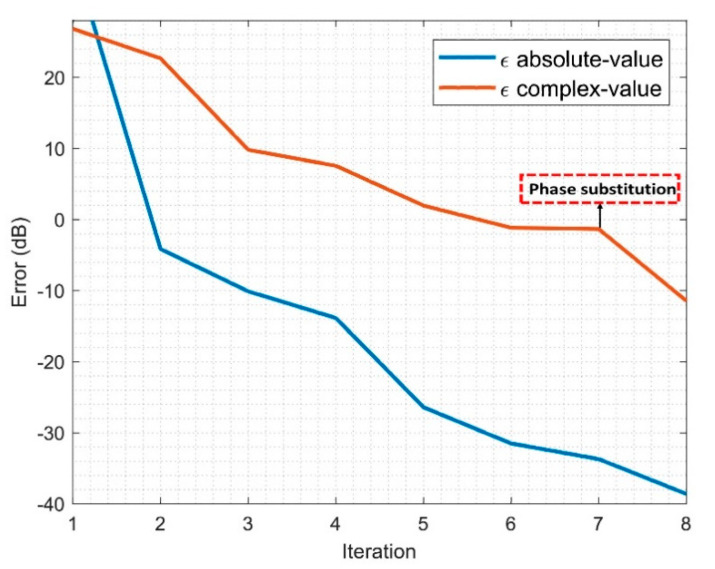
Iterative algorithm evolution.

To better understand the performance of the algorithm, the evolution of the error will be analyzed in terms of the complex and cost-function errors. The cost-function error will follow Equation (9), whereas the computation of the complex error (only possible in simulations) will follow Equation (11). The fields are phase normalized to the same point before the computation of the error since the solution retrieved can be multiplied by an arbitrary complex exponential of the form $e^{j\varphi_{0}}$. The evolution of the algorithm is depicted in Figure 8.

(11) $$\epsilon_{complex}={\left|Ax-b\right|}_{2}$$

It is well known that the cost-function error may be decreasing while the complex error is saturated. However, in the particular case of the algorithm proposed, modal filtering and phase substitution will help the algorithm to find a better starting point in the next iteration. Therefore, after this step, the algorithm convergence is improved.

So far, only the power spectrum and error evolution has been analyzed. If good convergence is found in the spectrum, a low error will be found in the reconstructed fields. In that sense, another example will be analyzed to show the NF and FF errors that can be expected in noiseless scenarios for electrically medium antennas. In the following results, the number of multiprobe arch positions corresponds to three. The minimum sphere radius r_0_ will be fixed to 8λ and d to 5λ.

**Figure 9:**
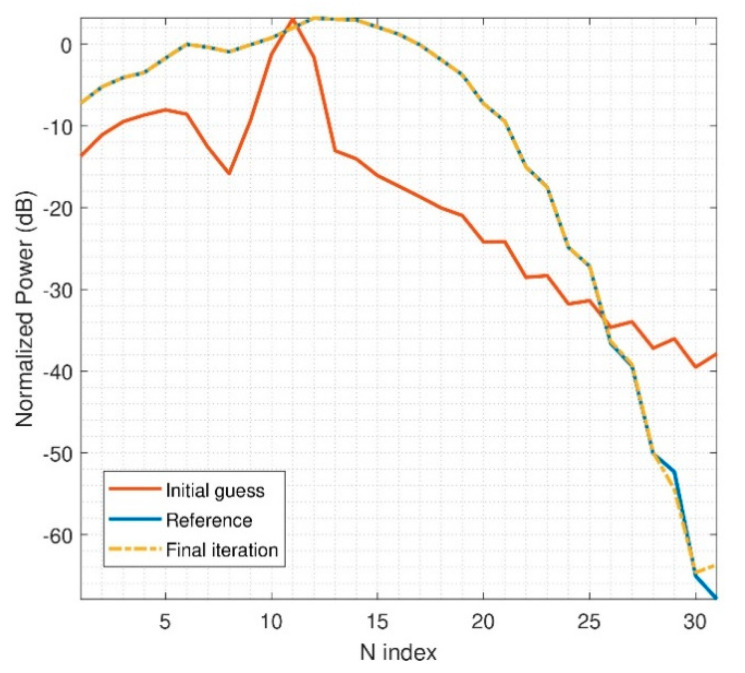
N-power spectrum of reconstructed field, r_0_ = 8λ, d = 5λ, 3 arch rotations.

**Figure 10:**
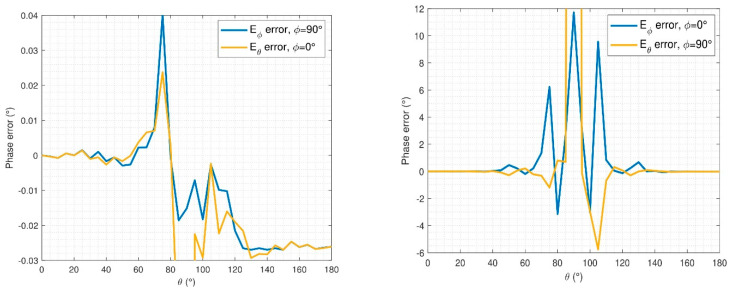
Near-field (NF) retrieved phase error for E_θ_ and E_φ_.

It is expected for the initial guess of this simulation to be even worse than the one obtained for the previous examples. In Figure 9, the power spectrum of the initial guess, reference, and final iteration are compared. It is observed that for three positions of the arch, the algorithm is able to find a low-error solution, which will be translated into low phase reconstruction errors. In Figure 10, the phase error of the retrieved NF is depicted for the tangential components of the reconstructed field. The computation of this error is done according to Equation (12), where the phases are normalized with respect to the same point to avoid constant phase errors. As expected, there is a very good agreement in the reconstructed NF phase.

(12) $$\epsilon_{\phi }=\phi_{reconstructed}-\phi_{ref}$$

The error analysis of the reconstructed FF from the iterative process showed the good agreement found between the simulations and the reconstruction. The FF mean error for Eθ and Eφ components is −95 dB and −91 dB, respectively, which represents the good performance of the algorithm for noiseless scenarios. 

## 4. Noisy Numerical Simulations

In this section, the performance of the algorithm will be tested when noise uncertainties due to the receiver are included. The noise model that will be used here is based on the model of a low-cost receiver that was already presented in [26]. These low-cost receivers represent a practical and versatile low-cost solution for EMC or over the air (OTA) measurements. In the following simulations, the relative phase and amplitude of each sampling point will be contaminated by noise due to the SNR of the reference and probe channels. The errors in each sampling point are introduced according to Equation (13), where *Xi* refers to a Gaussian distribution with 0 mean and standard deviation equal to 1, whereas ***σ|A|*** and ***σφ*** represent the standard deviations in amplitude and phase. The empirical validation of this model was already done and used in [26].

(13) $$\vec{E}=\left(\left|\vec{E}\right|+\sigma_{\left|A\right|}\cdot X_{1}\right)e^{j\left(\phi +\sigma_{\phi }\cdot X_{2}\right)}$$

The following considerations are followed when introducing errors in the sampling points:

- It is assumed that the noise floor of the receiver is 60 dB below the maximum of the NF acquisition.
- The simulated field is divided into N subsets, and each subset error is computed separately, depending on the Signal to Noise Ratio (SNR) of the top probe for each subset and azimuth position.

Once the errors are introduced by following the previous considerations, the first step from Figure 4 is performed in order to obtain the initial guess. This process is not error-free anymore and when projecting the fields according to Ludwig’s third definition, the error introduced in the reference and probe channel will be reflected into errors between azimuth cuts. 

**Figure 11:**
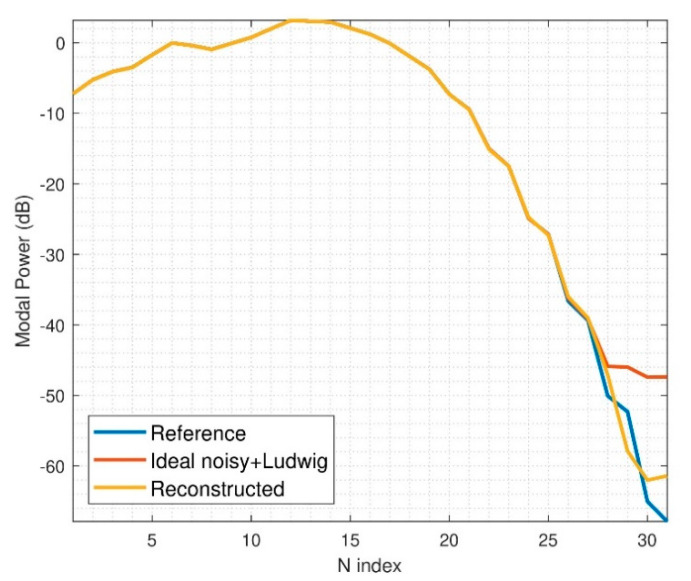
N-power spectrum for noisy simulation, arch rotation of 3.

For comparison purposes, one of the simulation examples from Section 3 will be evaluated. In particular, three arch rotations are simulated, with a minimum sphere radius of 8λ and d equal to 5λ. In Figure 11, the output spherical power spectrum of the reconstructed field is compared with the ideal one. The ideal one refers to the spectrum that would be retrieved if only errors due to the first phase retrieval process are present, which is when the top probe elevation rotations are not introducing errors. Obviously, in this simulation, the linear combinations will give information perturbed by noise, as well as the phase substitution step. However, the modal filtering will make the iterative algorithm to find solutions where high-order modes are not very prominent, and this represents an advantage.

**Figure 12:**
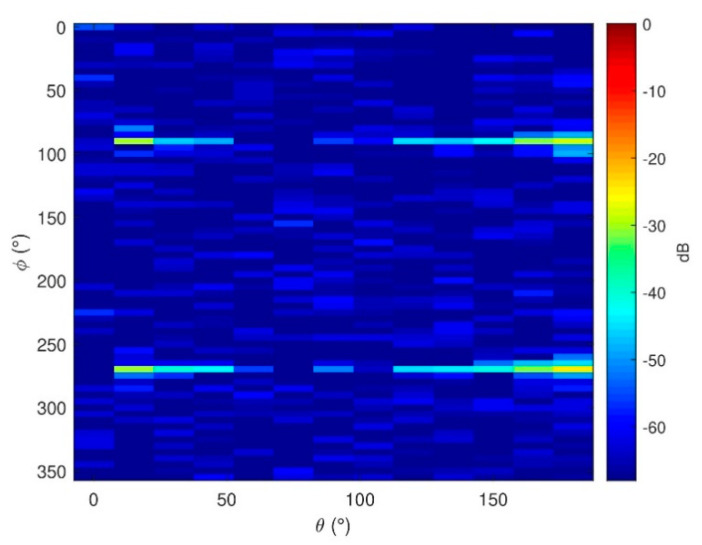
Electric NF error of Eφ for the first subset after the initial phase retrieval.

The error introduced by the first phase retrieval process can be seen in Figure 12. It is observed that for this simulation, the errors are mainly located around φ = 90° and φ = 270°, where the SNR of the top probe of the first subset is lower. The phase information of these cuts could be avoided since they represent information with a high uncertainty. Nevertheless, it is not implemented here, and the results obtained represent the performance of the algorithm by following the phase variations including errors due to the top probe for all azimuth cuts, even the ones where the SNR is very low. 

**Figure 13:**
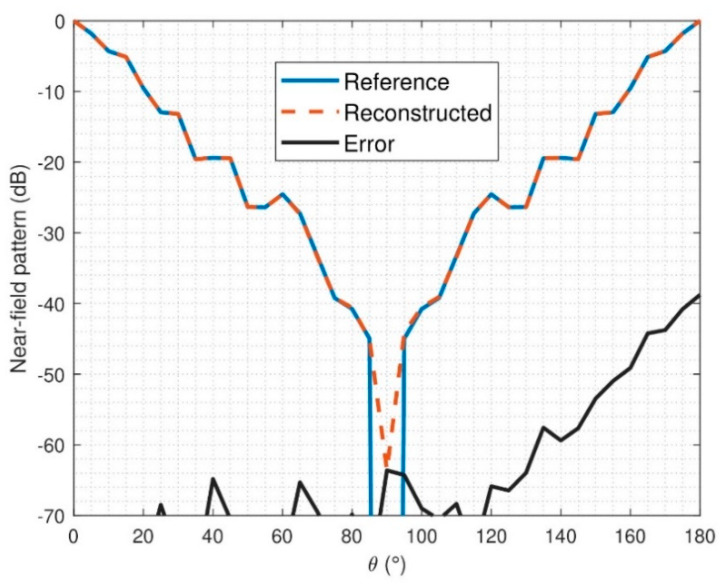
NF comparison of reference and retrieved electric field for noisy simulation, E_θ_, φ = 0°.

**Figure 14:**
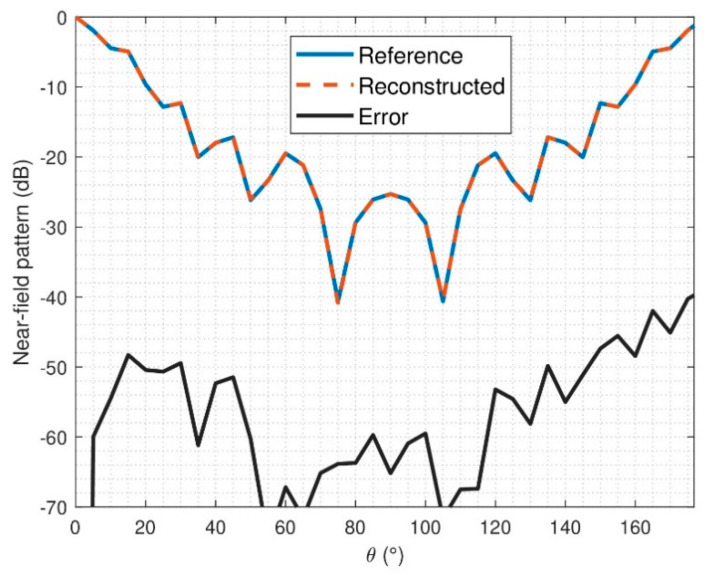
NF comparison of reference and retrieved electric-field for noisy simulation, E_φ_, φ = 0°.

The NF and FF pattern errors have also been analyzed and computed according to Equation (14). In the NF case, the errors are depicted in Figure 13 and Figure 14. It can be observed that the reconstruction obtained is not as good as it was for the ideal case; this was already inferred from the modal analysis of the reconstructed field. Indeed, it can be observed that small constant phase errors are present in the reconstruction from θ ∈ [90°, 180°] in both components, E_θ_ and Eφ.

(14) $$\epsilon (\mathrm{dB})\;=20\log_{10}\left|{\vec{E}}_{reconstructed}\;-{\vec{\;E}}_{reference}\right|$$

The mean FF error of the reconstructed field is −72.2 dB and −73.4 dB for E_φ_ and E_θ_, respectively. The results show the good convergence of the algorithm, even including errors intrinsic to the noise due to the top probe technique and receiver. 

## 5. Measurement Results

In this section, the iterative algorithm is tested on measured data. The data is acquired in the classical manner, which is amplitude and phase measurement. Then, the effect of measuring a self-transmitting device is included by multiplying the known phase by random errors. In that way, the changes of the reference channel during a real measurement of a self-transmitting device is emulated.

The spherical multiprobe measurement data of a SH2000 antenna [27] at 2 GHz will be used as input for the reconstruction algorithm. The measurement system used for the acquisition was the MVG system StarLab [28]. The StarLab angular step between probes is 22.5°. For this measurement, it was necessary to perform three arch rotations, that means 7.5° sampling step since the same sampling is used in elevation and azimuth. The following steps were followed in order to reconstruct the electric field:

1. The measured field (amplitude and phase) of the SH2000 is divided into three subsets (three different multiprobe arch positions).
2. For each azimuth cut of the different subsets, the electric field is multiplied by a random phase. In that way, it emulates the effect of measuring with a reference channel that is changing between azimuth cuts.
3. The subset where the top probe is on-axis for all the different azimuth cuts is used in order to compute the initial guess.
4. Then, the iterative algorithm is used to determine the phase unknowns introduced by the arch rotation.
5. Finally, the reconstructed field is compared with the reference one. The reference corresponds to the measured field of the SH2000 without any phase distortion due to the reference channel effect.

In this particular case, the error introduced by the reference channel when the signal to noise ratio is compromised is not analyzed. This error was already studied in [21]. Nevertheless, it was demonstrated that this error is not critical since the error is only concentrated around one azimuth cut where the reference signal to noise ratio is low. Moreover, in most practical measurements, the signal to noise ratio is enough in order to minimize the introduced errors. Thus, the error introduced in the computation of the initial guess can be considered as negligible. 

**Figure 15:**
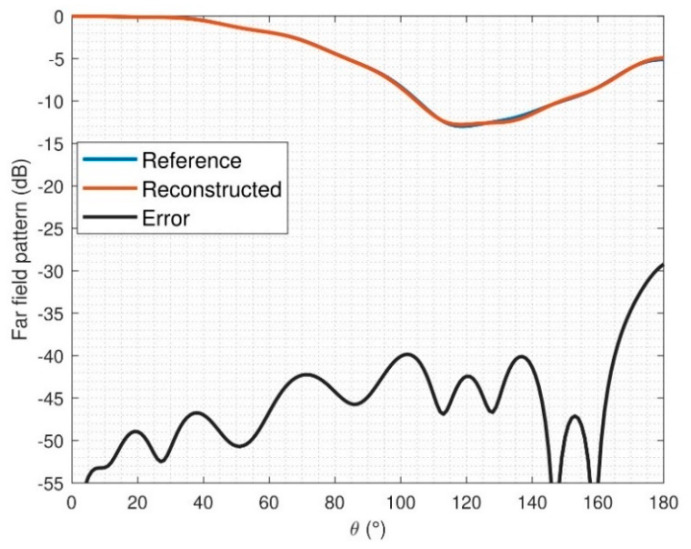
Far-field (FF) comparison after reconstruction of SH2000 for E_φ_, φ = 0°.

**Figure 16:**
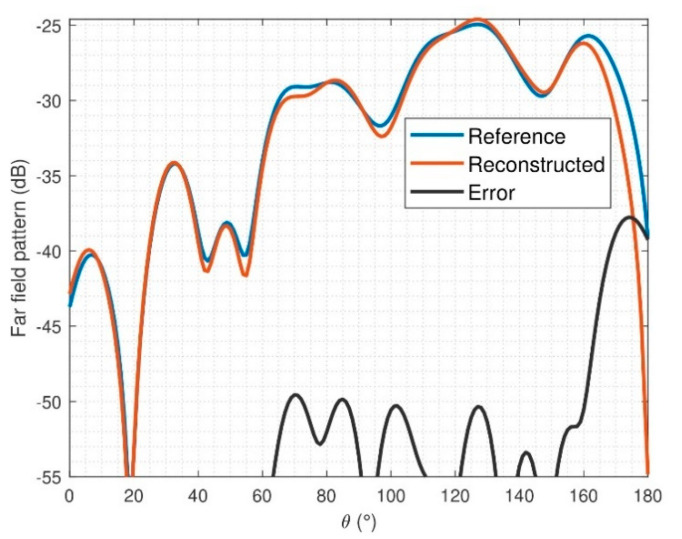
FF comparison after reconstruction of SH2000 for E_θ_, φ = 0°.

The number of N-index modes used to compute the spherical expansion of the antenna was 20, which means that the AUT represents a good trade off in terms of antenna size and processing time with the optimization algorithm. The agreement between FF patterns and errors of the reconstructed field for φ = 0° can be seen in Figure 15 and Figure 16. As it was mentioned above, the reference radiation pattern is the measurement of the SH2000 with the StarLab system. The mean error for Eθ and Eφ is −51.3 dB and −48.9 dB, respectively. 

## 6. Conclusions

In this paper, an iterative optimization algorithm to retrieve the phase of self-transmitting devices in spherical multiprobe measurement systems has been presented. The solution is suitable to address the characterization of devices derived from 5G or massive wireless networks between other scenarios. The approach herein presented is based on a linear combination of measured signals at different positions. The top probe of the multiprobe arch is considered to be the reference channel of the measurements. For fixed multiprobe arch scenarios, the phase differences can be reconstructed by appealing to Ludwig’s third definition. However, when the multiprobe arch is rotated to increase the number of samples, the iterative optimization algorithm is necessary to incorporate the a priori information of the relative phase between measurement points. Therefore, the field is subdivided into subsets and an iterative algorithm is applied by using as initial guess the output of the phase reconstruction on the first subset. The computational complexity of the algorithm makes it suitable for electrically small or medium problems. Some numerical examples have been analyzed in this paper for different sizes and multiprobe schemes. In noise-free scenarios, the results obtained show that the reconstruction errors are very low. In order to achieve these errors, the initial guess for different iterations of the algorithm is improved by applying modal filtering and phase substitution. A real error model of a low-cost receiver has been used to simulate the errors introduced during a measurement due to the SNR of the measurement probes and the reference channel. For these cases, the initial guess may not be that good, but the results obtained with the algorithm can even improve the input phase differences due to the modal filtering between iterations. The measurement results show the good agreement that can be found in practical scenarios after the reconstruction process.

Future work of this paper concerns the optimization of the proposed algorithm to cover electrically large devices. Furthermore, the use of advanced post-processing techniques like non-uniform discrete Fourier transform (NUDFT) in combination with the proposed algorithm could be interesting to face irregular grid measurements as the ones derived from robotic arm solutions. 

## Data Availability

Not applicable.
